# Health policy and systems research agendas in developing countries

**DOI:** 10.1186/1478-4505-2-6

**Published:** 2004-08-05

**Authors:** Miguel A Gonzalez-Block

**Affiliations:** 1 Manager Alliance for Health Policy and Systems Research World Health Organization CH 1211 Geneva 27, Switzerland

## Abstract

**Background:**

Health policy and systems research (HPSR) is an international public good with potential to orient investments and performance at national level. Identifying research trends and priorities at international level is therefore important. This paper offers a conceptual framework and defines the HPSR portfolio as a set of research projects under implementation. The research portfolio is influenced by factors external to the research system as well as internal to it. These last include the capacity of research institutions, the momentum of research programs, funding opportunities and the influence of stakeholder priorities and public opinion. These dimensions can vary in their degree of coordination, leading to a complementary or a fragmented research portfolio.

**Objective:**

The main objective is to identify the themes currently being pursued in the research portfolio and agendas within developing countries and to quantify their frequency in an effort to identify current research topics and their underlying influences.

**Methods:**

HPSR topics being pursued by developing country producer institutions and their perceived priorities were identified through a survey between 2000 and 2002. The response to a call for letters of intent issued by the Alliance in 2000 for a broad range of topics was also analyzed. The institutions that were the universe of this study consisted of the 176 institutional partners of the Alliance for Health Policy and Systems Research producing research in low and middle income countries outside Europe. HPSR topics as well as the beneficiaries or issues and the health problems addressed were content analyzed. Topics were classified into 19 categories and their frequency analyzed across groups of countries with similar per capita income. Agendas were identified by analyzing the source of funding and of project initiation for projects under implementation.

**Results:**

The highest ranking topic at the aggregate level is "Sector analysis", followed by "Disease burden" and "Management and organization". Categories at the bottom of this ranking are "Equity", "Policy process", "Economic policy and health" and "Information systems". "Disease burden" is more often funded than other topics for which there is more demand or perceived priority. Analysis suggests few although important differences across priorities, demand for funding and actual project funding. The donors' agenda coincides most with the ranking of research topics overall.

Ranking across country income groups shows important differences. Topics that gain prominence in low income countries are "Disease burden" and "Accessibility". In lower middle income countries "Insurance" gains prominence. In upper middle income countries "Decentralization/local health systems", "Equity" and "Policy process" are more prominent. "Program evaluation" is the most consistently ranked topic across income regions, showing a neutral influence by donors, governments or researchers.

**Conclusions:**

The framework proposed offers a basis to identify and contrast research needs, projects and products at the international level and to identify the actor agendas and their influence. Research gaps are suggested when comparing topic ranking against the challenges to health system strengthening and scaling up of disease control programs. Differences across per capita income groups suggests the need for differentiated priority setting mechanisms guiding international support. Data suggests that stakeholders have different agendas, and that donors predominate in determining the research portfolio. High-level consensus building at the national and international levels is necessary to ensure that the diverse agendas play a complementary role in support of health system objectives.

The Ministerial Summit for Health Research to be held in Mexico in November 2004 should be an opportunity to analyze further data and to commit funding for priorities identified through sharing and discussion of agendas.

## Background

Countries and international agencies have made a qualitative leap in the funding of the global disease challenges. The Global Fund for AIDS, TB and Malaria has received pledges totalling over US$ 2 billion. Bilateral donors are also making important funding contributions. In this context, strengthening of health systems has become a critical issue. Research can play a major role to identify the best policies to channel massive efforts, to ensure that vertical approaches do not fragment fragile health systems and to monitor and evaluate progress. How relevant is the research effort being undertaken in developing countries, and how is the agenda being driven?

WHO is organizing the Ministerial Summit on Health Research, to be held in Mexico City, 23 to 26 November, 2004. The main theme will be the role of health research in meeting the Millennium Development Goals. Health policy and systems research (HPSR) will have a prominent role in the context of the scaling up of efforts against major diseases and child and maternal mortality. Looking towards the Summit, WHO established a task force to identify HPSR priorities as an effort to advocate for major funding in this area. The Alliance for Health Policy and Systems Research, an initiative of the Global Forum for Health Research in collaboration with WHO, has been promoting since its inception in 2000 the identification of research priorities among producer institutions in developing countries.

This paper proposes a conceptual framework and a methodology to think about the HPSR research portfolio, the agendas that influence it and the priority setting process. The paper makes the case for the formulation of HPSR priorities at national, international and global levels. A previous article [1] looked at research capacity among Alliance partner institutions in the South and identified the need to increase funding to establish long term research projects as a basis for sustainable capacity building. Using data from the same survey, indicators are proposed to assess the priority setting process on the basis of various types of data. The scope and value of the conceptual framework and its indicators are illustrated by presenting the HPSR topic ranking on the basis of Alliance partner contributions and the influence upon it of country income and actor agendas. A forthcoming paper will present findings from a new survey now being completed and covering the universe of research producers in the South.

### Conceptual framework

Priority setting efforts are often bogged down because of inadequate methods of categorization of the research that is undertaken and the influences shaping it. These influences, in turn, are often not adequately understood, including the nature and role of priorities. The conceptual framework proposed here strives to offer some simple definitions of the research portfolio and its various influences, as well as indicators to measure and relate these concepts.

The nature of overall health research has been defined in terms of basic, applied and strategic research. These categories are useful to guide investment decisions which might maximize benefit [2,3]. However, within the field of health policy and systems research, little effort has been given to the classification of what is studied. Before priorities can be identified, it is important to be able to agree on what is studied through an analysis of the dimensions that characterize the object of research. Furthermore, it is important to establish the distinction between the object of the research and the factors shaping this choice at various levels.

#### The HPSR portfolio

To identify the object of research the concept of the research portfolio can be useful. The HPSR portfolio can be defined as the current set of research projects on health system structures, functions, processes and results at national, international and global levels. Projects, as distinct from plans or priorities, would include the commitment of resources towards a specific, time-bound aim and a set of objectives.

It has been proposed that the research portfolio should be analyzed along three dimensions of strategic importance: where to make investments, that is, the identification of the object of research or thematic areas in which investments are made; the type of investment research and development (R & D) instrument used and the resources spent through each area and type of instrument [3]. While our contribution aims to develop the first dimension, it is useful to expand on the other two to understand their interrelations.

The Ad Hoc Committee on Health Research proposed three types of R & D instruments: discovery oriented research to develop new health products and interventions; innovation research to adapt efficacious but unaffordable interventions to make them cost-effective, and implementation R & D to achieve greater efficiency in the use of existing interventions [2]. Harrison has argued for the need to consider a fourth instrument of equity R & D to ensure that the research portfolio responds to the poor and the underlying health problems in developing countries.

With regard to funding, there is a need not only to consider investments, but also funding sources and mechanisms. Four broad types of sources can be recognized: bilateral and multilateral donors, government commissioning, private commissioning, and funding through resources available to research institutions as part of their budgets. Each will have different implications for the kind of knowledge produced and for its possible influence on the health system [1]. This subject has been explored for Alliance partners in developing countries, identifying the amounts and sources of funding for their research portfolio [4].

#### The object of research

Previous analyses have revealed a complex heterogeneity along which researchers classify HPSR in developing countries, which is not surprising in an interdisciplinary field [5]. However, five overarching dimensions can be recognized:

• concepts reflecting the health system, such as policy and financial structures, regulatory functions, processes such as technology evaluation and quality monitoring, and results such as satisfaction and health gain

• the levels of the health system, such as the households and the community, first level facilities and hospitals

• the issues or problems pertaining to the health system such as priorities, equity and the public private mix

• the populations addressed by the system, such as children, mothers and the elderly, or rural and urban populations

• the health needs addressed, whether in terms of risks or disease.

While these dimensions can be useful to characterize the research portfolio, it is clear that there will be overlaps; for example, equity is both an issue and an attribute of the health system, particularly if it has been integrated in monitoring and regulation. In order to make use of these dimensions it is proposed to consider as the project topic the first dimension of concepts pertaining to the health system structures, functions, processes and results. The topic could then be classified following normative or theoretical frameworks or by using the categories researchers apply in their own research. The other four dimensions can be used to qualify the research topic as to provide a more detailed description. These four dimensions could be selectively used or aggregated to facilitate description according to the needs at hand.

#### Analysis of portfolio characteristics

Each of the five project dimensions can be analyzed in terms of the range of items considered. The ranking and emphasis of each item can also be revealed by analyzing its frequency. A research portfolio at any level can be very focused and comprise a narrow set of topics. Or it can be wide-ranging across many health system functions, structures, processes and results. It has been argued that research portfolios should be more focused on improving immediate health problems through operations research in low income countries, where funding and human resources are very limited [3]. However, the challenges of scaling up disease control programs call for research at the health systems level also.

#### The level of the research portfolio

The level at which the portfolio is analyzed is important, as it will have different characteristics and uses. At national level, the HPSR portfolio would be the set of projects addressing health and health system problems within the confines of national borders and governmental authority as well as sector-wide and inter-sectoral issues. Examples would be the impact on equity of decentralization policies, or the roles played by conflicting policy actors in scaling up of services. The international research portfolio would be topic areas which are common across a number of countries or regions. Identification of the international portfolio could serve, among other purposes, to fund research at regional or international levels, to strengthen the critical mass of research available to inform country policy making and lesson learning across countries, and to extend the range of methodological approaches through comparative research [6]. The HPSR portfolio at global level refers to research themes and projects that are, by their very nature, supra-national. This would involve, for example, international financing of immunization efforts, intellectual property rights in health research, and development of international disease control measures.

#### The HPSR agendas

The HPSR portfolio at any level is influenced by factors within and outside the research system [7]. Within the research system the following factors can be identified: research capacity; research trends and preferences expressed by researchers and research institutions; research funding and market opportunities; and research preferences voiced by policy makers, service managers and public opinion. Outside the health system the broad factors shaping the portfolio are the health conditions and health system problems as well as the cultural, economic and political context. As a whole, these factors shape actor-specific research agendas that express ethical, professional and political values and that influence the allocation of scarce resources towards alternative project portfolios.

It is clear that if actor agendas have few areas in common there will be no consensus and therefore no overall priorities. Significant overlap of interest, on the other hand, can lead to the identification and formulation of shared research priorities. Research priorities are therefore defined as the explicit areas of agreement on, and ranking of, the object of research across diverse actor agendas. Priorities can then become policy instruments to coordinate diverse agendas towards a common end without forcing a single research agenda.

The characteristics of agendas and of priorities can be identified through the same kinds of dimensions and indicators as the research portfolio. That is, preferences can be classified in terms of topics, issues or beneficiaries, and health problems. The characteristics of the HPSR agendas and priorities can also be studied in terms of the range of topics, issues, levels, populations and health problems. They can also be ranked and their emphasis revealed by frequency analysis. In this way, the agendas can be compared across themselves, priorities can be identified as common topics and issues and with similar ranking and emphasis, and the influence of agendas and priorities on the actual research portfolio can be assessed.

#### Priority setting through agenda co-ordination

It has been argued that co-ordination of the various influences shaping the HPSR portfolio can increase the impact of research on equity and can contribute to its strategic role for development [8]. Co-ordination would involve developing a consensus of researcher, policy maker and investor agendas. Such a consensus should ideally result in a highly coherent set of topics across the various actors' research agendas and, eventually, a high degree of correspondence between agendas and the research portfolio.

HPSR portfolio change through co-ordination would involve a gradual process of adjustment to new priorities, project completions, maturation of research capacity and funding opportunities. Coordination requires interfaces and mechanisms such as Research Forums and Essential National Health Research Mechanisms [9] to develop a consensus while allowing, and even encouraging, critical differences.

In sum, the HPSR portfolio can encompass a differing range of topics with diverse rankings and emphases; can be more or less coherent with respect to researcher, funding and policy maker agendas; and can be more or less co-ordinated along a set of shared priorities. The interplay of these dimensions can give rise to a number of scenarios, of which three are here illustrated.

a) Co-ordination could lead to focusing the HPSR portfolio on a few, highly cost-effective topics in a situation of few resources and well identified, high priority needs. There would be, eventually, a high degree of coherence across the portfolio and agendas held by various actors. The risks here would be lack of diversity to foster innovation and healthy criticism.

b) In a situation of plentiful resources the research portfolio and its driving agendas could be wide-ranging and have little overlap. Specific portfolio segments would correspond with particular agendas, thus satisfying multiple interests. However, co-ordination through overarching mechanisms could ensure integration of knowledge around high level priorities. In this manner a unified, although highly diverse, HPSR field of enquiry would be obtained.

c) In a situation of lack of coordinating mechanisms, with low resources, the portfolio could focus on a reduced set of topics, each satisfying a particular agenda and thus fragmenting resources and hindering support to health system development. If resources are more plentiful, lack of coordination could lead to a rich but highly dispersed and inefficient research portfolio with little impact on development.

Irrespective of the availability of resources, co-ordinating mechanisms are likely to be important to ensure an efficient use of research resources.

### Methodology and Indicators

Two sources of information were used to illustrate the conceptual framework proposed. The first was a survey of Alliance-HPSR partner institutions in developing countries detailing research priorities and project information. Researchers reported here on the priorities they had received from policy makers in the course of diverse consultations in the past year. The second source was a database of letters of intent (LOI) submitted to the Alliance for funding, where projects were justified on the basis of priorities negotiated by researchers and policy makers or service managers.

#### Content analysis

This proceeded in several steps. A preliminary list with 24 research topics was identified through an inductive analysis using the research statements expressed in the LOI, which were the most detailed. This list was then used to classify research topics in the projects and priorities expressed in the Alliance partners' profiles. Beneficiaries/issues and diseases/health problems addressed by the LOI, projects and priorities were also categorized and classified at this stage. While projects may have contained more than one topic or beneficiary/issue, the most prominent one was selected. In a few cases where several topics were considered this was indicative of a sector-wide analysis and classified accordingly.

The beneficiaries/issues of the research were classified to include any of four alternative dimensions: a) the demographic group: elderly or children/adolescents, b) level of care: community, primary or hospital, c) the geographical focus: urban or rural, and d): gender, equity/poverty, indigenous populations, and public-private mix. Whenever more than one dimension or aspect was applicable (which occurred only in a small number of statements), a decision was made to include the most prominent.

The concept "equity" was classified both as a topic and as an issue. It was assigned as a topic whenever equity was the main objective of the research and it was addressed through a number of health system attributes such as financing, access, and service delivery. Equity/poverty was considered as an issue when the poor were identified as the main subjects of research or when the equity implications of research directed mainly to another topic were highlighted as a major concern.

The distribution of statements was analyzed according to country income group: low income, lower middle income and upper middle income. These income groups correlate highly with geographical regions, with LI being mostly in Africa and Asia, LMI mostly in Asia and with a particular weight by China, and UMI in Latin America and the Caribbean (table 1).

**Table 1:** Glossary of Health Policy and Systems Terms Used for Content Analysis

**TOPIC**	**TERMS FOUND IN RESPONSES**
Accessibility	Health seeking behaviour, determinants of utilization, coverage, outreach, referral, barriers to care, willingness and capacity to pay, cost-sharing, price regulation, prices, equity in access, demand for health services.
Community participation	Community-based strategies, community participation in governance, empowerment, school health, family health strategies, social support networks.
Costing & cost effectiveness	Determination & evaluation of costs, cost-benefit of services, economic evaluation, cost-effectiveness of resource allocation, alternative uses for resources.
Decentralisation/local health systems	Decentralization policy and process, impact of decentralization on services and health outcomes, district health system development, healthy cities, municipal health services, local government, devolution, community participation in local health services.
Disease burden	Prevalence and incidence of diseases, mortality and morbidity, disease profiles, health status, health needs, burden of disease studies, risk factors, determinants of health and disease other than economic or social policy.
Economic policy and health	Free trade agreements and health, TRIPPS and health, economic crises and health, impact of poverty reduction and adjustment policies on health, debt reduction and health, social policy and health, social assistance and health issues, intersectoral co-ordination, labour policies and health.
Equity	Equity of health system, impact of health reforms on equity, equity and poverty, poverty targeting of services, poverty and health, exclusion.
Financing	Financial mobilization, financial allocation, financing policies, national & district health accounts, financial equity, community health financing, financing of specific programmes.
Human resources	Personnel management, deployment, migration, motivation, knowledge, attitudes and practices of health personnel, satisfaction, quality of life, human resource policy, human resource performance, traditional healers, training and education of human resources, medical education curriculum assessment, evaluation of medical and nursing teaching programmes.
Information, education and communication (IEC)	Information and communication for the general public, health education strategies and impacts, knowledge attitudes and practices (KAP).
Information systems	Information needs, informatics, surveillance mechanisms and systems, strengthening of information systems, health monitoring systems, establishment of public domain databases, development of indicators for service management and policy.
Insurance	Risks and benefits covered by insurance schemes, community based health insurance, options for health insurance, insurance reform, impact of insurance on health and service outcomes.
Management & organization	Health service provider performance, delivery of services, administration, service management strengthening, contracting and provider payment mechanisms, impact of privatization on services, performance agreements, impact of hospital autonomy on service delivery, stakeholders in service management, community participation in management.
Pharmaceutical policy & management	Rational drug use, procurement, logistics, herbal medicine, dispensing practices, pharmaceutical regulation, national drug policy, essential lists.
Policy process	Stakeholder analysis, role and relationships of actors in the formulation and implementation of policy, role of government agencies in policy formulation, role of community and NGOs in policy formulation, factors influencing policy process, perceptions of policy, decision-making processes, policy negotiation.
Programme evaluation	Evaluation and assessment of impact of policies or programmes on specific diseases or services.
Quality	Clinical practice guidelines, evidence-based medicine, quality assurance, patient satisfaction.
Research to evidence	Health systems research training, health systems research training, outcomes of research, research impact, policy utilization and impact of research, research methods, creation of national HPSR database, priority setting of health research, research ethics, essential national health research, dissemination of research.
Sector Analysis	Health sector reforms and implications, health systems development, private health service development, intersectoral collaboration and co-ordination, public/private mix health care, health care organization, regulation, policy formulation on specific diseases, on programmes or on aspects of the health system, sector-wide and system-wide performance.

Two researchers classified all statements independently and disagreements were discussed and resolved. The 24 topic categories were reduced to 19 to avoid groups with less than 2% of the total number of statements while maintaining topic coherence. Table 1 presents the glossary of terms included under each topic.

The frequency of responses by country for all types of statements is generally proportional to country population, with China, India, Brazil and Bangladesh at the top of the frequency. However, countries with a strong health systems research presence are over-represented, such as Colombia, Argentina, Philippines, Thailand, South Africa, Uganda, Ghana, Cuba, Costa Rica, Benin, Jamaica and Tanzania.

### Identification of agendas

**Figure 1:**
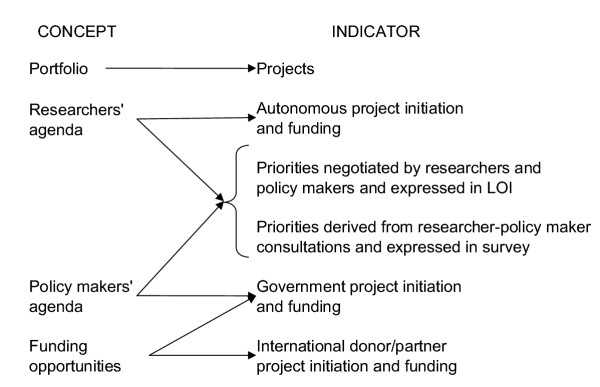
Concepts and indicators

The range and emphases of the HPSR portfolio and agendas were mapped through topic content and frequency analysis (Figure 1). Project and agenda data were also aggregated from the two sources to obtain a general mapping of topics. This was used to assess coherence across actor agendas and with the portfolio and to increase the number of observations to enable analysis by income level.

Two methods were used to assess the coherence between the HPSR portfolio and the agendas held by researchers, policy makers and international donors and partners. The first method used the survey data to infer the agendas by observing the topic frequency of: projects proposed and funded by researchers without external assistance; projects initiated and funded exclusively by government, and projects initiated and funded exclusively by international stakeholders or research partners.

The second method compared the research portfolio against the agenda expressed by policy makers. The policy maker agenda was observed through the survey as reported by researchers and through the LOI as negotiated with researchers. A negligible influence by the donor, in this case the Alliance-HPSR, would be expected in the LOI given that the call requested priorities within the generic definition of HPSR presented above.

Each of the three modes of identification of agendas could have method-specific biases. In the first method, preferences are derived from the portfolio itself, that is, from research projects in implementation. Furthermore, the method isolates the preferences expressed by each actor. As such, this method could be deemed to reveal most objectively preferences behind each actor funding or initiating a project. However, projects under implementation may hide topic preferences that are not translated into projects or topics that were generated and funded through joint actor participation.

The observation of negotiated priorities expressed in LOI captures the mix or balance of researcher-side influences and policy maker needs. It will therefore reflect a consensus position across each actor. However, it will exclude the influence of funding opportunities, will not reveal actor-specific preferences and will be limited by the constraints placed on the LOI (see below).

Priorities based on consultations between researchers and policy makers and expressed by researchers through a survey will reveal the understanding and conceptual framework of researchers and may underplay policy-maker needs. Furthermore, these priorities will be influenced by the research projects under execution and reported in the same instrument.

#### Assessment of coordination between portfolio and agendas

The analysis of relationships or influences across the portfolio and each of the actor agendas, as well as of similarities or differences between agendas, was undertaken by correlating topic frequencies across lists and by undertaking a qualitative analysis of changes in rank order and emphasis.

Analysis of the range and rank of topics across groups of countries by income was undertaken by aggregating project and priority topic data into a reference list representing the combined set of influences on the agenda-setting process, including the portfolio itself. The aggregation of data into a reference list was mainly a strategy to increase the observations and make the analysis more reliable, although it may have validity if it describes the overview of the agenda-setting factors at play. That is, the actual portfolio can be conceived as a force shaping the agendas, together with other factors.

#### Survey and LOI database

The survey of HPSR producer institutions in developing countries was described in detail elsewhere [1] and includes information for 108 of the 176 Alliance-HPSR partners (61% response rate) who produced research in low and middle income countries outside Europe between 2000 and 2001. The database contains information on the current research portfolio (294 projects were declared) as well as research priorities (402 priorities were stated, with a maximum of 5 per survey). Information on project initiation and source of funding is available for 270 projects. A total of 39 developing countries out of a total of 133 were contacted. Respondents are close to one sixth of the close to 650 institutions known to the Alliance to be producing HPSR in developing countries.

Biases in the partner database could have occurred as a result of preferences by certain type of institutions in joining the Alliance HPSR and in answering the questionnaire required from partners. Over-representation at both levels could have occurred of more competitive and productive institutions with larger project portfolios and funding, and more interest in international funding. On the other hand, larger institutions may have been discouraged from responding given the larger number of projects to be reported, although they would also have more capacity to respond. Furthermore, the response rate could have been lower among institutions where producing HPSR is not a main function.

The LOI database has 403 submissions for research funding in response to a call by the Alliance-HPSR in 2000. Applicants requested funding for one year projects in high priority areas identified jointly by them and national policy makers and stakeholders. A limitation of this database is the exclusion of funding requests for projects over one year as well as topics that would be formulated solely by researchers. Analysis of the partners' database indicates that 24% of projects are of longer duration and that up to 34% of projects undertaken are initiated by the research institution without stakeholder collaboration.

#### Expansion and standardization

The frequencies of statements for each income region were expanded proportional to population to make comparisons across regions possible. The frequency of statements across the three types of statements (projects, LOI and priorities) was standardized to give each equal weight when aggregating them to analyze the combined representation of the research portfolio and the agenda-setting process as a whole.

Responses show a distribution across income regions proportional to population in some cases and with significant biases in others (table 2). The low income region (LI) has 50% of the population and 47% of statements, while the upper middle income (UMI) region has only 12% of the population but twice the number of statements, with 22%. The lower middle income (LMI) region is also somewhat under-represented, with 38% of the population and 31% of statements.

**Table 2:** Distribution of Statements According to Type, Content Category and Geographical Region by Income Group

	**LI**		**LMI**		**UMI**		**TOTAL**	**%**
			
**Types of statements**	**Total**	**%**	**Total**	**%**	**Total**	**%**		
Priorities	198	49	143	36	61	15	402	100
Letters of intent	193	48	101	25	109	27	403	100
Projects	124	42	97	33	73	25	294	100
**TOTAL**	**515**	**47**	**341**	**31**	**243**	**22**	**1099**	**100**
**Content categories**								
Topics	482	46	330	31	237	23	1049	100
Beneficiaries or Issues	217	53	113	28	80	20	410	100
Health Problems	132	58	48	21	48	21	228	100
**Total statements by geographical region**								
Africa	247	77	15	5	57	18	319	100
Asia	260	53	214	44	13	3	487	100
Latin America and the Caribbean	8	3	112	38	173	59	293	100
**TOTAL**	**515**	**47**	**341**	**31**	**243**	**22**	**1099**	**100**
Total population in Geographical region		50		38		12		100

The frequency of statements on priorities is as would be expected for the population in each region. However, project statements and demand for funding are biased in favour of UMI, with 25% and 27% of the statements, respectively, against 12% of population share.

#### Ranking

This was done for each topic or category within the topic by rounding percentage differences to integers and grouping in the same rank all categories falling within the same percentage.

## Results

This article does not attempt to provide an exhaustive analysis given the fact that data is limited to Alliance partner producer institutions in the South. The purpose here is to illustrate the potential of the proposed methodology and to present the most robust findings. HPSR topics are first presented and analyzed aggregating in a single list the topics in the research portfolio as well as in the policy maker and researcher agendas. This aggregate representation is then analyzed by groups of countries according to their per capita income. The influence exerted on the HPSR portfolio by various actors is then analyzed.

### Characteristics of HPSR producers in developing countries

HPSR producer institutions are generally small with an average of 3 projects, 8 researchers and a project portfolio worth $155,226 [1]. Only 19% of researchers have a PhD qualification, although researchers in key disciplines are well represented and better qualified. Research capacity and funding are similar across income regions, although inequalities are apparent.

### Overview of topics

A total of 19 research topics were identified when aggregating portfolio (project) and priority (voiced preferences) data into the reference list. Topics ranged in frequency from 2% to 11% and were ranked in 8 classes (Table 3). The highest ranking topic is "Sector analysis" with 11% followed by "Disease burden" with 9% and "Management and organization" with 8%. From here three topics rank lower equally at 7%, two rank at 6%, seven rank at 4% and then two each at 3% and 2%. Categories at the bottom of this ranking are "Equity", "Policy process", "Economic policy and health" and "Information systems". The emphasis of topics at the top end is then about five times as greater as those at the bottom end of the range.

**Table 3:** Ranking of Topic at the Aggregate Level

**Rank**	**Topic**	**%**
1	Sector Analysis	11
2	Disease burden	9
3	Management & organization	8
4	Accessibility	7
	Programme evaluation	7
	Research to evidence	7
5	Financing	6
	Human resources	6
6	Community participation	4
	Costing & cost effectiveness	4
	Decentralisation/local health systems	4
	Information, Education and Communication	4
	Insurance	4
	Pharmaceutical policy & management	4
	Quality	4
7	Equity	3
	Policy process	3
8	Economic policy and health	2
	Information systems	2
**Rank**	**Beneficiaries/Issue**	**%**
1	Community	15
2	Equity/poverty	14
3	Hospital	12
4	1st level	11
	Gender/women	11
	Rural areas	11
5	Children/adolescents	10
6	Public private mix	6
7	Urban areas	5
8	Elderly	4
9	Indigenous peoples/traditional medicine	3
**Rank**	**Health Problem**	**%**
1	Reproductive health	30
2	HIV-AIDS	11
	Nutrition	11
	TB	11
3	Chronic	7
	Environmental health	7
	Malaria	7
	Mental health	7
4	Other infectious	6
5	Other	3

The fact that "Equity" appears so low in the aggregated ranking could be partly attributable to the fact that this topic was defined to include only projects and priorities having equity as the central topic and measuring it through multi-dimensional approaches such as health conditions, access to services and financing. A subsidiary analysis was thus undertaken to include under "Equity" those projects or priorities addressing equity or poverty as a secondary, qualifying, role of research on other topics. This broadened topic "Equity" climbs to fourth rank, at the same level as "Accessibility", "Program evaluation" and "Research to policy".

Public and private institutions show no significant changes in topic ranking (corr = 0.70). "Community participation" and "Accessibility" are the only topics with major differences, ranking higher among private institutions.

### Topic analysis by beneficiary/issue

Out of the total topics classified in the reference list, only 38% (404) were sufficiently focused or detailed to be able to attribute a beneficiary or specific issue (Table 4). This was mainly the case with priority statements, which by their very nature were generic. The beneficiary or issue statements were spread across the 11 categories identified through content analysis. The category with least statements had only 2% of the total, and that with most 13%.

**Table 4:** Beneficiaries/Issues According to Topic

	**Beneficiaries or Issues**																							
			
**Topic**	Elderly		Children		Community		Primary		Hospital		Urban		Rural		Equity		Gender		Indigenous peoples/traditional medicine		Public private mix		TOTAL	
	
	A	B	A	B	A	B	A	B	A	B	A	B	A	B	A	B	A	B	A	B	A	B	***n *= **	*B*
Accessibility			2	3	10	7	7	7	10	8	7	12	19	**20**	17	14	19	17	2	11	7	7	42	10
Community participation	9	**20**	4	3	**43**	19	13	7			13	12	4	3			13	7					23	6
costing & cost effectiveness			13	3	13	2			**38**	6	13	4	13	3	13	2							8	2
Decentralisation/local health systems			13	5	**27**	7	7	2			13	8	27	10	7	2	7	2					15	4
Disease burden	15	**50**	**21**	19	3	2	3	2	6	4	12	15	9	8	6	4	**24**	17	3	11			34	8
Economic policy and health	14	10	**43**	8											14	2	**29**	4					7	2
Equity	6	10			6	2									**78**	**28**			11	**22**			18	4
Financing					**39**	17					4	4	9	5	**39**	18	4	2			4	2	23	6
Human resources					**29**	9	**35**	15	6	2	6	4	18	8					6	11			17	4
Inform. Educ. & Communication			**21**	8			7	2	7	2	14	8	14	5			**36**	11					14	3
Information systems					**25**	4	**38**	7	13	2			13	3	13	2							8	2
Insurance					**44**	13					13	8	19	8	13	4			6	11	6	2	16	4
Management & Organization			2	3	9	7	9	10	**49**	**48**	2	4	11	13	2	2	4	4			13	14	47	12
Pharmaceutical policy & Mgmnt.							17	5	**33**	8			8	3	**25**	6	8	2	8	11			12	3
Policy process					**50**	6					17	4			**33**	4							6	1
Programme evaluation	2	10	**22**	24	2	2	17	17	5	4	12	19	2	3	2	2	2	2			**32**	**30**	41	10
Quality					7	2	**20**	7	**47**	15			7	3			13	4			7	2	15	4
Research to evidence							33	2							**33**	2					**33**	2	3	1
Sector Analysis			11	11			11	10					8	8	11	8	8	7	3	11	**47**	**40**	36	9
None			**26**	14	5	2	11	5					5	3			**47**	**20**	5	11		0	19	5
n		10		37		54		41		48		26		40		50		46		9		43	404	100
%	**2**	100	**9**	100	**13**	100	**10**	100	**12**	100	**6**	100	**10**	100	**12**	100	**11**	100	**2**	100	**11**	100		

A = % across the row ; B = % down the column

The topics with least identification of beneficiary or issue were "Costing and cost effectiveness", "Policy process" and "Research to Evidence", with 79% to 96% in this situation. By contrast, "Community participation" and "Management & Organization" were the topics least frequently unidentified with beneficiary/issue, at between 30% and 41%.

The most frequent beneficiary/issues were Community, Equity/poverty Hospital, Gender Primary care and Rural areas. The three beneficiary/issues least identified were Urban areas, elderly and Indigenous peoples/traditional medicine.

The analysis of the correlation between beneficiary/issue and topic is tentative at this stage given the low frequency in many of the cells of the 19 by 11 matrix. Two beneficiaries/issues account for a large part of the focus of research topics: Community and Hospital as a focus on levels of care. At the community level the topics of community participation, financing, health insurance, decentralization, policy process, information systems and human resources are all prominent. At the hospital level the topics of costing and cost effectiveness, pharmaceutical policy, quality of care and management and organization are most prominent. By contrast, the topic of program evaluation is fairly widely spread across several issues or beneficiaries.

The following topics show also a fairly discreet relationship to beneficiaries/issues: Research on accessibility is mostly focused on rural areas. Research on disease burden is prominent among the elderly and children. Economic policy and health focuses on children. Gender is also an important component of these three topics. Equity is focused on indigenous populations. The topic of information, education and communication is prominent among children. Sector analysis focuses mainly on the public private mix.

### Topic analysis by income level

The differences in ranking of the topics in the reference list across income regions are shown in the first three columns of Table 5. Larger differences occur in 9 topics, mostly in lower middle income and upper middle income countries. This suggests that the reference list reflects more closely lower income country needs. Largest differences were observed in health insurance, decentralization/local health systems and, equity and policy process, topics that are more highly ranked in upper middle income countries.

**Table 5:** Ranking of Topics in the Reference List and Differences by Income Region and by Project Initiation

RANK	**TOPIC**	**INCOME REGION**			**PROJECT INITIATION**		
		
		Low	Lower Middle	Upper Middle	Donors	Govnt.	Research Institution
1	Sector Analysis				--	-	
2	Disease burden					--	
3	Management & organization		+				--
4	Accessibility	+			+	--	--
	Programme evaluation						--
	Research to evidence			-		-	+
5	Financing	-	++			++	--
	Human resources		+				
6	Community participation			-	+	+	++
	Costing & cost effectiveness	--	+		+	+++	
	Decentralisation/local health systems	-		+++		+	
	Information, Education and Communication				+		
	Insurance	--	+++	-			+
	Pharmaceutical policy & management						
	Quality					+	
7	Equity	-		+++	+		+++
	Policy process	-		+++			
8	Economic policy and health	-	+		+		+
	Information systems				+	+	

Cells with a rank difference of 1, 0 or -1 are blank. Changes of 4 or more = +++; 3 = ++; 2 = +; -2 = -; -3 = --; -4 or more = ---

### Comparing the research portfolio and the agendas

The overall ranking of topics in the reference list was compared against the ranking of topics in projects initiated by each actor. The relationship between actor preferences and the reference list was assessed through an analysis of their rank congruence.

As described in a previous paper in more detail [1], the research institution is the initiator in 34% of projects, while 31% are initiated by a donor agency, international research partner or by a private contractor. Governments initiate in 24% of cases. 12% of projects are reported as a mix of the above and are not considered for this analysis.

The agendas across actors differ substantially, and none can be said to be close to the other. As a result, the reference list shows marked difference with respect to each actor's agenda. Government initiation shows preference for financing and cost-effectiveness as compared to the overall ranking. Government initiation tends to give lower regard to disease burden studies and for research on accessibility. International donor initiation matches best the reference list but gives somewhat less preference to sector-wide analyses. Research institution initiation is more marked for equity and community participation and less so for management and organization, accessibility and program evaluation. International donor initiation preferences are associated to the top ranking topics in the overall listing, suggesting a predominance of their agenda on the reference list.

## Conclusions

The analysis of the research portfolio and priorities at the international level shows a widely diversified set of topics, ranging from sector wide issues to more focused program evaluation. The emphasis on sector wide issues reflects the challenges to health systems today and suggests that countries consider as important the macro-level analysis as the micro. Micro approaches with a focused attention to beneficiaries or specific issues are well identified, particularly under the topic of program evaluation.

However, the evidence also suggests a gap between the research that is actually being undertaken and the challenges for strengthening and scaling up of disease control programs. Such a gap is evident in the low emphasis given to research on human resources, policy process, equity, economic policy and health and information systems.

By contrast, the analysis suggests a high degree of attention at the community level, although much attention is also given at the hospital level. Primary care thus seems to be under-emphasized. Considering the disease focus, whenever this was made evident, the data do not suggest a bias towards problems that would not be evidently important at country level.

The fact that the public or private character of research institutions is insignificant for the agenda suggests the capacity of diverse institutions to work within a common agenda.

There are significant differences in the research portfolio across groups of countries based on per capita income, suggesting the need for priority setting mechanisms at both national and international levels that reflect such diversity. The greater congruence between donor preferences and the international research agenda highlight the importance of consensus building between national and international actors. While it is appropriate for governments and international donors to fund different aspects of the research portfolio, this requires high-level priority setting and consensus mechanisms to ensure they complement each other rather than lead to fragmentation.

More research is required to establish the relationships between actors' agendas and the research portfolio at the international level. There is also a need to discuss the most desirable balance of influences and to increase the voice of developing country actors. Evidence-based HPSR priorities emerging through such a process would then be able to support scaling up of research efforts on a par with scaling up of health system strengthening and disease control. Regional and global meetings, such as the Ministerial Summit for Health Research to be held in Mexico in November 2004, are good opportunities to present and discuss the evidence and to commit funding accordingly. Attention must be given to encouraging consensus building on research priorities within regions comprising countries with similar needs. The interests of donors, governments, health workers, the community and researchers must all be taken into consideration so that research funding leads not only to fund relevant research but to build the necessary interfaces for utilization.

## Competing interests

None declared.
